# Barriers and motivations for health insurance subscription in Cape Coast, Ghana: a qualitative study

**DOI:** 10.1186/s13690-017-0192-x

**Published:** 2017-05-29

**Authors:** Akwasi Kumi-Kyereme, Hubert Amu, Eugene Kofuor Maafo Darteh

**Affiliations:** 1 Department of Population and Health, University of Cape Coast, Cape Coast, Ghana; 2 Department of Population and Behavioural Sciences, School of Public Health, University of Health and Allied Sciences, Hohoe, Ghana

**Keywords:** Health insurance, Motivation, Barrier, Subscription, Theory of planned behaviour

## Abstract

**Background:**

One of the main objectives of the Ghana National Health Insurance Scheme, at its establishment in 2003, was to ease financial burden of the full cost recovery policy, particularly on the poor. However, currently, majority of the scheme’s subscribers are individuals in the upper wealth quintile, as the poor in society rather have not subscribed. We explored the motivational factors as well as the barriers to health insurance subscription in the Cape Coast Metropolis of Ghana.

**Methods:**

This study collected qualitative data from 30 purposively selected subscribers and non-subscribers to the National Health Insurance Scheme using an in-depth interview guide.

**Results:**

Major motivational factors identified were; affordable health insurance premium, access to free drugs, and social security against unforeseen health challenges. Encouragement by friends, family members, and colleagues, was also found to motivate subscription to the health insurance. The major barriers to health insurance subscription included; long queues and waiting time, perceived poor quality of drugs, and negative attitude of service providers both at the healthcare facilities and the health insurance office. The study underscores the need for the National Health Insurance Authority to conduct intensive education to change the negative perception people have regarding the quality of health insurance drugs. Efforts should also be made to reduce the waiting time in accessing healthcare with the National Health Insurance Scheme card. This would motivate more people to subscribe or renew their membership.

**Conclusions:**

The implication of barriers found is that people may not subscribe to the scheme in subsequent years. This would, therefore, consequently defeat the objective of achieving universal healthcare coverage with the scheme.

**Electronic supplementary material:**

The online version of this article (doi:10.1186/s13690-017-0192-x) contains supplementary material, which is available to authorized users.

## Background

Challenges in financing healthcare are major issues of public health concern to governments and international health organizations across the globe [1]. For most nations, these challenges are mainly due to weaknesses in policies for financing healthcare. Strategies aimed at addressing issues related to international health policy have, therefore, consequently dominated the debate and health research on the global front [2] with governments instituting various interventions including health insurance policies to improve the health of their residents.

All over the world, there are two main approaches to financing of health insurance schemes by countries; private and public/government [3]. Private health insurance is often paid by individuals [3]. Some employers in the private sector, however, contribute to the private health insurance schemes of their employees [4]. In some countries, households contribute to community-level financing schemes, which pool monies that are managed on behalf of all members of the private scheme [4]. Public/government health insurance is generally paid using general tax revenue. In many developing countries, external aid and grants from donor partners also constitute 20–80% of funding for public health insurance schemes [4]. Private and public approaches of financing health insurance schemes often co-exist in many countries [3]. For instance, community-level insurance schemes usually complement public schemes, while private medical insurance may receive tax breaks which amount in practice to public/government subsidies [3].

While health insurance schemes have seen improvements in subscriber base, years and decades after their introduction in some countries, the health insurance schemes of other countries either made no improvements or experienced retrogression in number of subscribers [5–7]. Colombia, for instance, approved its Universal Health Insurance Scheme (Law 100) in 1993 [8] which created the National Social Security System for Health (NSSSH), and currently covers more than 95% of the Colombian population. The approved healthcare policy was a universal health insurance scheme that entitled all citizens of the country, irrespective of their ability to pay, to a comprehensive healthcare benefits package [9].

Prior to 1993, the spread of public health insurance was relatively limited in Colombia with only 24% of the population covered; a quarter to a third of the country’s population [8, 9]. When the Universal Health Insurance Scheme, which is a public health care financing scheme, was introduced in 1993, however, a 49% subscription rate was recorded in that same year [8]. By 2007, 70% of Colombia’s population had subscribed to the health insurance and by 2011, more than 95% of the Colombian population were covered under the SGSSS (Sistema General de Seguridad Social en Salid) [9]. Colombia achieved a universal coverage with health insurance by the year 2012 [10].

The Universal Coverage Scheme (UCS), which is Thailand’s version of public health insurance, was implemented in 2001 [11]. This, according to Sakunphanit and Suwanrada [12], consolidated earlier public health insurance schemes which were targeted at improving access to healthcare among the poor in the country. These other insurance schemes were Civil Servants Medical Benefit Scheme (CSMBS), Social Security Scheme (SSS), Medical Welfare Scheme (MWS), and Health Card Scheme (HCS) [11]. Like Colombia, Thailand met its full implementation target of covering over 95% of its total population by 2012 [11–14]. Social security as used in the present study refers to the measures put in place by governments and international organisations such as the United Nations Organisation, that guarantee access to sufficient food and shelter resources, as well as promotion of health and wellbeing for all.

Taiwan implemented its National Health Insurance (NHI) in 1995 [15]. The scheme was introduced as a public universal health insurance programme, which was meant to cover comprehensive healthcare services for all residents [15]. Prior to implementation of the NHI, Taiwan’s healthcare providers were paid by clients out-of-pocket. Also, before the NHI was introduced in 1995, about 57% of Taiwan’s population were insured through three separate social health insurance schemes [15]. These were, government employee insurance, labour insurance and farmers insurance [16]. Implementation of the NHI, however, astoundingly saw 96% of the population subscribing to the health insurance by the end of 1995 [16]. By the end of 2001, 97% of the total eligible population of Taiwan had subscribed to the NHI [17]. The implication is that like Thailand and Colombia, Taiwan has been successful in implementing health insurance.

Unlike Thailand, Colombia and Taiwan, Nigeria’s National Health Insurance Scheme, which is also a public health insurance scheme, (NHIS) has not been a success story [11]. In efforts to ensure that every Nigerian has access to good healthcare services, the NHIS of the country introduced various programmes to cover different segments of the Nigerian society [11]. These are; formal sector social health insurance programme, urban self-employed social health insurance programme, rural community social health insurance programme, children under-five social health insurance programme, permanently disabled persons social health insurance programme, prison inmates social health insurance programme, tertiary institutions and voluntary participants social health insurance programme, and the armed forces, police and other uniformed services health insurance programme [18]. However, since its launch in 1999, the National Health Insurance Scheme covers only about 3% (5 million subscribers) of the country’s total population [11].

Even though the NHIS in Ghana was promulgated in 2003 (National Health Insurance Law [Act 650 of Parliament]), mandatory insurance, had a legal backing in 2004 (National Health Insurance Regulations [L.I.] 1809) [19, 20]. The NHIS is a public health financing scheme which seeks to provide healthcare to all persons resident in Ghana irrespective of nationality, race, sex and age.

Financing of the scheme is done with 2.5% of Valued Added Tax (VAT) on goods and services as insurance levy, yearly premiums paid by persons 18 years of age and above, 2.5% deductions from pension contributions of workers in the formal sector with the Social Security and National Insurance Trust (SSNIT) [21]. The NHIS is also funded through monetary allocations made to the Health Insurance Fund (HIF) by parliament (Ghana’s legislature) in addition to donations, grants, gifts, investments, voluntary contributions [2]. Pensioners under SSNIT, those who are at least 70 years, pregnant women, and children under 18 years of age, however, form exemptions on the scheme from payment of yearly premiums [21].

The NHIS covers about 95% of the disease burden of Ghana [21]. These encompass services provided for out-patient clients such as diagnostic testing and operations including hernia repair; most in-patient client services which consist of care by specialists, majority of surgeries and accommodation at the wards of health facilities; services related to maternal care including caesarean sections; treatments for oral health; emergency care; and drugs listed on the medicines list of the scheme [5].

The body authorised by law to manage the NHIS is the National Health Insurance Authority (NHIA). The NHIA, in efforts to improve the scheme’s performance in meeting the health needs of Ghanaians, has since its inception in 2003, taken a number of initiatives. These include the introduction of a free maternal health care in 2008, creation of a clinical audit in 2010, establishment of a claims processing center in 2010, introduction of a consolidated premium account in 2011 and introduction of the NHIS call center in 2012 [22]. Biometric identification cards were also introduced by the NHIA in 2014 to help improve client identification as a way of enhancing efficient service delivery [21].

Although, one of the main objectives of the NHIS at its establishment in 2003, was to ease financial burden of the full cost recovery policy, particularly on the poor [5], majority of NHIS subscribers are individuals in the upper wealth quintile, as the poor in society are rather unable to subscribe to it [21, 23]. Full cost recovery was a health financing policy introduced in 1983 by the government of Ghana, where clients were made to pay for the entire cost of treatment whenever they accessed government health facilities and services [24]. Health insurance subscribers who are provided with poor services tend to be dissatisfied as they spend longer hours before they are attended to, compared with clients who access healthcare through payments out-of-pocket [25]. There is, therefore, the need to investigate the motivations for subscribing the health insurance, and the barriers which confront both current and potential subscribers.

Subscription to the NHIS has been studied by a number of researchers [21, 23–26]. These studies have, however, been conducted among scheme managers and service providers with little attention to subscribers. Attention has also not been paid to barriers faced by non-subscribers. Besides, most of them addressed health insurance subscription from a quantitative point of view. The present study, therefore, explores the barriers and factors that motivate individuals’ subscription to health insurance in the Cape Coast Metropolis among both subscribers and non-subscribers.

### Conceptual framework

**Fig. 1:**
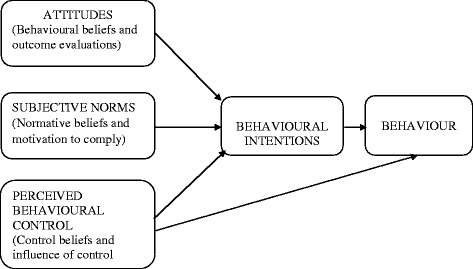
Theory of Planned Behaviour. Source: Ajzen [27]

The Theory of Planned Behaviour which was developed by Ajzen [27] was adapted as the conceptual framework for the study. It is a behavioural theory which predicts deliberate behaviour, because behaviour can be deliberative and planned [28]. The tenets of the theory are: attitudes, which comprise behavioural beliefs and outcome evaluations; subjective norms, which consist of normative beliefs and motivation to comply; perceived behavioural control which is based on control beliefs and influence of control beliefs; and behavioural intentions (Fig. 1). All of these tenets result in a particular behaviour, which in the case of the present study is subscription to health insurance [28, 29].

Attitude towards a behaviour entails an individual’s general assessment of a particular behaviour. It is assumed to have two elements which work together [30]. These are, beliefs regarding the consequences of the behaviour (behavioural beliefs) and the corresponding positive or negative judgements about each of these features of the behaviour (outcome evaluations) [31]. Subjective norms about a behaviour refer to an individual’s own estimate of the social pressure exerted on him or her to perform that particular behaviour (subscription to health insurance) [32]. Subjective norms have two components, which work in interaction [32]. These are beliefs about how other people who may be in some way important to the person, would like him or her to behave (normative beliefs) and the individual’s motivation to comply with these perceived expectations from those who may be in some way important to him or her [33].

Perceived behavioural control is the extent to which an individual feels capable to act out a behaviour (subscription to health insurance) [29]. This construct also has two elements which comprise the amount of control that an individual has over a behaviour (subscription to health insurance) and how confident he or she feels about being able to perform (subscribe) or not perform (not subscribe) the behaviour (health insurance) according to Ajzen [27]. Perceived behavioural control entails control beliefs regarding the power of both internal and situational variables to facilitate or militate against the performing of the behaviour (health insurance subscription) [28, 29].

**Fig. 2:**
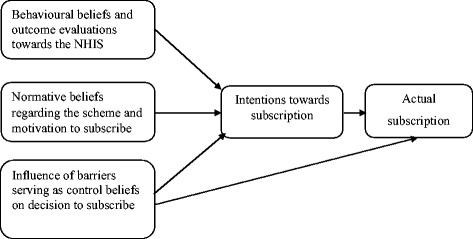
Conceptual framework. Source: Adapted from Ajzen [27]

The theory helped in explaining how behavioural beliefs and outcome evaluations, normative beliefs and motivation to comply as well as control beliefs and influence of control beliefs influence behavioural intentions regarding subscription to health insurance, which then consequently result in the decision of people to either subscribe or not subscribe, to the NHIS, in the Cape Coast Metropolis. For an individual to subscribe to the health insurance for instance, he or she assesses the barriers and motivations for subscribing. If the barriers outweigh the motivations, the individual is most likely not to subscribe to the scheme and vice versa (Fig. 2).

## Methods

Ghana is located in West African with a total land area of 238,533 km^2^ [34]. The country generally low-lying in exception of Mountain Afadjato which is the maximum point (883 m) above sea level, and a series of hills on the eastern border. According to the 2010 population and Housing Census of Ghana, the country had a population of 24,658,823 [33]. Of this, there were 12,024,845 males and 12,633,978 females. This implies that males formed 48.8% while females constituted 51.2% of the population. This thus resulted in a sex ratio of 95 males to 100 females. The country also has a population density of 103 per square km [33]. The main ethnic groups in Ghana are the Akans with 47.5%, Mole-Dagbanis with 16.6%, Ewes with 13.9%, Ga-Dangmes with 7.4%, Gurmas with 5.7%, Guans with 3.7%, Grusis with 2.5%, and Mandes with 1.1% and others with 1.4% [34]. Ghana has 50.9% of its population living in urban areas while 49.1% reside in rural areas. There are 3217 functional health facilities in the country. Out of this number, 4 are teaching hospitals. Also, there are 9 regional hospitals, 3 psychiatric hospitals, 11 polyclinics, 96 government/public hospitals, 59 Christian Health Association of Ghana (CHAG) hospitals, 22 quasigovernment hospitals, 10 Islamic hospitals, and 156 private hospitals. The majority of these health facilities are found in the urban areas [35]. Ghana’s Fertility has declined from 7 children per woman to 4 over the last 3 decades. The high fertility, combined with decreasing mortality, has resulted an intercensal growth rate of around 2.5% per annum since 1960 [35].

Cape Coast Metropolis lies within latitudes 50^0^07 North and 50^0^20 North of the Equator and between longitudes 1^0^11 West and 1^0^41 West of the Greenwich Meridian [36]. The Metropolis is bounded to the West by the Komenda-Edina-Eguafo-Abrem (KEEA) Municipality, to the East by the Abura-Asebu-Kwamankese District, to the North by the Twifu/Hemang/Lower Denkyira District and to the South by the Gulf of Guinea. According to the 2010 population and housing census of Ghana, the total population of Cape Coast Metropolis was 169,894 out of which 82,810 (48.7%) are males and 87,084 (51.3%) are females [36]. The people are mainly Akans and Christians in terms of Ethnicity and Religion respectively. The reported total fertility rate for the metropolis was 2.21 [36]. There are both private and government/public health facilities in the Metropolis. With regards to the public ones, the metropolis is mainly served by the metropolitan hospital with the teaching hospital serving mainly as a referral point. The University of Cape Coast (UCC) and Ewim Hospitals as well as Adisadel Clinic supplement efforts of the Metropolitan Hospital. There are 11 private health facilities in the Cape Coast Metropolis [36].

Data for the study were collected from 30 residents of the Cape Coast Metropolis through purposive sampling. Purposive sampling was adopted to ensure that 15 subscribers and 15 non-subscribers to the NHIS, were sampled for the study. The use of purposive sampling was also to ensure that only those 18 years and above were selected as research participants. This is because the least age for paid subscription to the NHIS is 18 years [22]. Respondents were interviewed in their homes. Prior to inclusion in the study, potential interviewees were asked to indicate if they were subscribers or non-subscribers and if they were at least 18 years of age.

An in-depth interview guide was used to collect data from the participants. The interview guide was divided into three sections; A, B, and C. Section A was used to collect data on the socio-demographic characteristics of the respondents. Socio-demographic characteristics considered were; age, sex, marital status, religion, ethnicity, and level of education. Section B focused on the motivations for health insurance subscription. Issues addressed in this section comprised affordability of the scheme’s annual premium, ability to save extra money for other needs, social security against unforeseen heath challenges, and encouragement from friends and relations to subscribe. The last section of the instrument was based on barriers to subscription. Issues addressed in this section were; perceived quality of drugs, duration of wait time and length of waiting queues, and attitude of service providers.

Data collection was done at places which were convenient for the respondents. These included their homes and work places. An audio recorder was used to capture the interviews and notes were made of relevant observations. Prior to the main data collection, the research instruments were pretested in the KEEA Municipality to ensure trustworthiness. It was also explained to the respondents that the information they made available to the researchers were not going to be identified with them or about persons whom information were provided. To ensure that data obtained during the data collection process were protected from unauthorised access and hence ensuring confidentiality and privacy of the data, voice recordings were locked with a computer programme called ‘my lockbox’. Notes taken were also typed and the soft copies equally locked in ‘my lockbox’. The hard copies were however, hidden from sight. Anonymity of respondents was ensured by using pseudonyms where necessary, instead of the real names.

Data collected from respondents during the in-depth interview, were analysed using content analysis. The conceptual type of content analysis was adopted. Themes and codes were developed, which formed the basis of the analysis. Statements of the respondents were presented as quotes to substantiate the views expressed.

## Results

### Socio-demographic Characteristics of Respondents

The respondents were aged between 18 and 59 years. Twenty-one of them were aged 20 to 29 years, while one was aged below 20 years. Twenty of the respondents had never been married while ten were married at the time of the study. Christians (29) and Akans (23) formed the majority in terms of religion and ethnicity respectively. All the respondents also had some form of formal education with 12 attaining tertiary education (Table 1).

**Table 1:** Socio-demographic Characteristics of Respondents

Characteristics	Frequency	Percent (%)
Age		
< 20	1	3.3
20–29	21	70.0
30–39	4	13.3
40–49	2	6.7
50–59	2	6.7
Sex		
Male	15	50.0
Female	15	50.0
Marital status		
Never married	20	66.7
Married	10	33.3
Religion		
Christianity	29	96.7
Islam	1	3.3
Ethnicity		
Akan	23	76.7
Ewe	5	16.7
Ga/Adangme	1	3.3
Mole-Dagbani	1	3.3
Level of education		
Primary	1	3.3
JHS/Middle school	7	23.3
SHS/O’ Level/A’ Level	10	33.3
Tertiary	12	40.1

Source: Field data, 2015

### Factors motivating health insurance subscription

The major factors which motivated both subscribers and non-subscribers were: affordability of the NHIS premium; free drugs; social security; and encouragement from friends, family members and colleagues. Regarding affordability, a subscriber said:

*“I subscribed (to the NHIS) because; sometimes you wouldn’t get money to go to the hospital. When this happens, because you are NHIS subscriber and have paid something little as premium, you would be able to visit the hospital and if anything at all, you will pay just a little token and they (health professionals) will attend to you”. –* ***Subscriber, Male, 24 years*** *.*

*A non-subscriber corroborated this saying:*

*“The NHIS makes it possible for you to visit the hospital and get treatment free of charge after paying the yearly premium which I see to be very cheap. Once you have your card, that’s all. You don’t need anything else. Just go there (health facilities) and the doctors would attend to you”. –* ***Non-subscriber, Male, 36 years*** *.*

Regarding free drugs, the respondents noted that subscription to the health insurance makes it possible to get some drugs free of any charges at the point of service delivery. A male non-subscriber for instance had this to say:

*Since individuals covered by the scheme have access to free drugs and other services, they are able to seek healthcare anytime. For instance, pregnant women are provided with free drugs and the cost of some lab tests are minimal. This, I think, is a good motivation to be a member [of the scheme]. –* ***Non*** **-** ***subscriber, Male, 27 years.***

*A male subscriber also indicated:*

*If you go to the hospital and you don’t have NHIS, you’ll be asked to buy drugs and so on. I therefore subscribed to the NHIS so that I will always get free healthcare if I fall sick anytime, especially the drugs which are free once you have your card.* ***– Subscriber, Male, 24 years*** *.*

Concerning the NHIS serving as a form of security, the respondents noted that subscription to the scheme secures the individual against unforeseen health challenges. The following quotes summarise their responses:

*That’s indeed true because*, *sickness comes at any time and I would not be informed as to when and where it will come. With the NHIS, I know that any moment that I fall sick I would not be worried since I have insurance to help me. It’s just like saving money for specific reasons. –* ***Subscriber, Male, 25 years*** *.*

*“It (NHIS) is good and serves as a source of security for those who are on it. It’s more or less like a safeguard of your life against future problems concerning your health. It secures you from any disease which may afflict you at any time. But as for me, I’ve signed an agreement with my bankers (Barclays Bank) and they do deduct from my monthly salary. This serves as a form of health insurance which when I fall sick, they use to cater for my expenses”. –* ***Non*** **-** ***subscriber, Male, 28 years.***

Encouragement from family, colleagues, and friends also motivated subscription to the NHIS. Respondents also indicated the reasons given by their family, colleagues, and friends for encouraging to subscribe or renew their membership. A non-subscriber for instance indicated that;

*Oh! As for that one, friends, family members, and some other people here (in the community) always tell me (to subscribe). My friends and family members for instance, normally say that I should get the card again so that when I go to the hospital, I’ll not have to pay anything. Aside that money they also said the card can be used for other purposes like identifying yourself and so I should get it.* ***– Non*** **-** ***subscriber, Female, 35 years.***

A female subscriber also said;

*My mother and my grandmother have always been encouraging me to remain being a member of the NHIS. They have been encouraging me to renew my card when it expires so that if I fall sick, I will be able to access healthcare without paying anything for it. They also say that if I don’t have the card, it will be difficult for them to support me financially when I fall sick.* ***– Subscriber, Female, 25 years.***

### Barriers to health insurance subscription

Perceived low quality of drugs was identified as a major barrier among both subscribers and non-subscribers. Non-subscribers for instance indicated that the poor quality of drugs given NHIS subscribers, was a major reason why they were not subscribed to the scheme. The following quotes summarize the experiences of the subscribers in this regard;

*In fact, the drugs that they give us (subscribers) in my opinion, are not of good quality at all. I am saying this because when you’re not feeling fine, maybe with malaria or something else and you go to the hospital, all they will just give you is para (paracetamol tablets). In fact! Para (paracetamol tablets) is constant. While I can buy para for just 30 Ghana pesewas, you give me para which doesn’t even work for Malaria and ask me to go and buy the other medicines outside. It’s very bad.* ***– Subscriber, Male, 24 years.***

*The quality of the drugs I was given the last time I went to the hospital was not good at all. You see, they (pharmacists) only give you para para para and that’s all. Then they’ll ask you to go and buy the rest elsewhere, even though they know para would not cure your sickness. They only told me that NHIS doesn’t cover the rest of the drugs and asked me to go and buy common vitamin C and that NHIS doesn’t cover it.* ***– Subscriber, Female, 28 years.***

*Non-subscribers also said;*

*Nowadays, if someone has NHIS and he or she goes to access healthcare, the doctors wouldn’t attend to him or her very well. Because the person has NHIS, the drugs that they will give him too wouldn’t cure his sickness at once. But if you pay out of your pocket, you’ll be attended to within a short time and every drug that you’ll be given too, will work very well for you. Also, sometimes, sickness that will normally take three days to treat, will take about six days, all because you are having insurance and the drugs given you are also not good at al. So to me, all these don’t serve as motivation for subscribing to the scheme.* ***– Non*** **-** ***subscriber, Male, 25 years.***

*The hospital I went to wasn’t a government hospital but a private one. They asked whether I had NHIS and I told them no, but they took care of me and gave me good drugs. The drugs really worked well for my sickness to go very fast when I took them. If I were an NHIS subscriber, the drugs that they would have given me, would have been nothing to write home about.* ***– Non*** **-** ***subscriber, Male, 25 years.***

Barriers identified among subscribers were: long queues and waiting time; and negative attitude of healthcare providers. While subscribers experienced long queues and waiting time, non-subscribers did not as there were usually two different queues at the healthcare facilities for out-patients with the longer one being for NHIS subscribers. A subscriber for instance said;

*Sometimes, the queues at the hospitals would discourage you from going to seek healthcare. Long queues at the NHIS office are also a major problem because, you will wake up early and go there, only for you to sit there and watch people who come late, go in and register and you will still be sitting there waiting to be attended to. I spent almost the whole day over there (NHIS office) the last time. Even if you have something to do at home, then you I’ll just be sitting there in the line doing nothing.* ***– Subscriber, Male, 32 years.***

*A non-subscriber, however, said:*

*It took me about two hours (to see a doctor). As for me, I was not carrying insurance card so, I was not in the longer queue. There were only some few people in front of me. So, the moment they finished I went to see the doctor. I think the 2 h I spent before seeing the doctor was okay because, those who came with the (NHIS) card, spent more time over there. There was this woman from my house for instance, who was there before I went to the hospital. When I was leaving, she was still in the queue.* ***– Non*** **-** ***subscriber, Male, 36 years.***

While attitude of healthcare providers particularly nurses, was negative towards health insurance subscribers, it was positive towards non-subscribers. A female subscriber for instance said;

*As for that one (attitude of nurses), I don’t even want to talk about it. Some of the nurses think they are better than anybody else they see. Even when you are talking to them, they don’t regard you as a client who deserves to be listened to. Rather, they start insulting you.* ***– Subscriber, Female, 27 years.***

*A male non-subscriber also indicated that;*

*Oh! The doctor who attended to me was normal (his attitude was positive). As for the nurses, you know they are always troublesome. But the last time I went to the hospital, none of them did anything negative towards me. Those who gave my medicines to me (pharmacists) also didn’t do anything bad to me or insulted me. I was only in the line (queue) and when it was my turn, they called me to come for my medicines.* ***– Non*** **-** ***subscriber, Male, 36 years.***

## Discussion

It was realised that one of the motivations for subscribing to the NHIS was affordability of the NHIS premium. This finding is consistent with the argument by Schultz, Metcalfe and Gray [37] that low premium of health insurance is the number one factor which motivates subscription to health insurance. The NHIS also served as a form of security against unforeseen health challenges which is consistent with the argument made by Mulupi, Kirigia and Chuma [38] that people see their subscription to the health insurance as a form of security against unforeseen health challenges/problems.

Long queues and waiting time were found as major barriers to NHIS subscription. These views corroborate findings by Mulupi et al. [38] that unnecessary delays in accessing healthcare due to long queues, usually serve as barriers to utilization of healthcare services and subscription to the health insurance. Perceived low quality of drugs given to NHIS subscribers was also realised as a major barrier to subscription. Drugs given to NHIS clients when they used health insurance to access healthcare were perceived to be of low quality compared to those given to the same clients when they accessed healthcare based on out-of-pocket payments. This finding confirms arguments made by Dalinjong and Laar [39] that perceived low quality of drugs received by health insurance subscribers when assessing healthcare is a major barrier associated with the insurance as seen by clients, as people are very often given different drugs when they report illness/disease conditions to hospitals with health insurance and with out-of-pocket payments.

The study also found attitude of service providers at the healthcare facilities to be negative. The findings are in line with suggestions by Arin and Hongoro [11] and Mladovsky and Mossialos [40] that attitude of service providers is negative and hence, serve as a major barrier to subscription to the health insurance and its renewal.

For participants who did not subscribe to the NHIS, we argue that their barriers outweighed the motivation for subscribing as noted by Wilper, Woolhandler, Lasser, McCormick, Bor and Himmelstein [41] in a previous study. Thus, barriers such as long queues and prolonged waiting time, and negative attitude of service providers towards subscribers, outweighed motivations including free drugs and the scheme serving as a form of security against ill-health [38].

The conceptual framework of the study posits that attitude towards a behaviour entails an individual’s general assessment of a particular behaviour and constitutes beliefs regarding the consequences of the behaviour and the corresponding positive or negative judgements about each of these features of the behaviour [30]. It was obvious from the findings that the judgements of the participants were negative particularly in relation to drugs given to the subscribers and waiting time. This is because, most of them indicated that the level of quality of drugs given the NHIS subscribers were low; an indication of negative perception towards quality of NHIS drugs and duration of waiting time.

Subjective norms regarding subscription were the pressures exerted on both subscribers and non-subscribers by both family and friends for them to subscribe or renew their subscription [32]. It was, however, realised that even though family and friends motivated non-subscribers for instance to subscribe, the subjective norms formed were not in favour of subscription [28].

It was realised from the study that some of the participants subscribed to the scheme to safeguard themselves against unfortunate health situations. This relates to the perceived behavioural control tenet of the conceptual framework [27], which posits the extent of control that an individual has over a behaviour (subscription to health insurance) and how confident he or she feels about being able to perform (subscribe) or not perform (not subscribe) the behaviour. The implication for the present study is that that some of the respondents, especially the subscribers internally assessed the barriers and motivations for subscribing to the scheme and opted to subscribe. After joining the scheme and experiencing so many challenges, however, it is likely they would not subscribe to it in future as suggested by Duku, Fenenga, Alhassan and Nketiah-Amponsah [42]. This is possible, especially if they realise the barriers to subscription outweigh the motivators.

Based on the findings made, intensive education should be carried out by the NHIA to change the negative perception people have regarding the quality of NHIS drugs. This will help to reduce the perception of low quality of drugs given to subscribers. Efforts should also be made by the NHIA to reduce the waiting time in accessing healthcare with the NHIS card to motivate more people to subscribe or renew their membership. This could be in the form of creating more offices to ensure a redistribution of the clientele at the various offices. Moreover, regular workshops should be organised by the NHIA and the Ghana Health Service to entreat their workers to exhibit positive attitudes towards clients in the exercise of their duties, so as to motivate more people to subscribe to the scheme.

Despite the significant findings made by our study, it is worth mentioning its possible limitations. Thus, the fact that the study was conducted in one city and used a relatively small sample size greatly limits its generalizability. This does, however, not limit its reliability and validity in any way.

## Conclusions

The implication of long queues and waiting time, perceived low quality of NHIS drugs, and negative attitude of service providers both at the healthcare facilities visited and the health insurance office is that people may not subscribe to the scheme in subsequent years. This would consequently defeat the objective of achieving universal healthcare coverage with the scheme.

## Additional file

Additional file 1: Interview guide for NHIS subscribers and non-subscribers. (DOC 42 kb)

## Abbreviations

CSMBS: Civil Servants Medical Benefit Scheme
HCS: Health Card Scheme
MWS: Medical Welfare Scheme
NHI: National Health Insurance
NHIA: National Health Insurance Authority
NHIS: National Health Insurance Scheme
NSSSH: National Social Security System for Health
SSS: Social Security Scheme
UCS: Universal Coverage Scheme
