# Supplementary material for: Barriers and motivations for health insurance subscription in Cape Coast, Ghana: a qualitative study
Source: Arch Public Health. 2017 May 29;75:24. doi: 10.1186/s13690-017-0192-x (PMC5447305; doi:10.1186/s13690-017-0192-x)
Supplement: Additional file 1: — Interview guide for NHIS subscribers and non-subscribers. (DOC 42 kb) [file 13690_2017_192_MOESM1_ESM.doc]

**Additional file 1**

**UNIVERSITY OF CAPE COAST**

**DEPARTMENT OF POPULATION AND HEALTH**

**INTERVIEW GUIDE FOR NHIS SUBSCRIBERS AND NON-SUBSCRIBERS**

Interview no: _______________________

Date of interview (DD/MM/YY): _______/_______/________

Time of interview: Start ________End________

Interviewer: ______________________

**SECTION 1: Background Characteristics of Respondents**

1. Age (In completed years)__________
2. Sex ______________
3. Marital status ______________
4. Religion___________________
5. Ethnicity __________________
6. Level of education___________

**SECTION 2: Factors motivating subscription to the NHIS**

1. (For subscribers only) What is/are your main motivation(s) for subscribing to the NHIS?

(For non-subscribers only) What are the main factors which may motivate an individual to subscribe to the NHIS?

1. How would you describe the yearly premium paid by NHIS subscribers? (Probe for the premium being either affordable or expensive).
2. What is your position on the assertion that the premium paid, enables an individual to save extra money which otherwise would have been spent on health care and thus serving as a motivation to subscribe to the scheme?
3. What is your position on the fact that the NHIS provides a form of financial protection against unforeseen health challenges?
4. What else do you see as motivating about the NHIS which may influence your decision to subscribe to the scheme/ renew your membership? (Probe for specific aspects of the scheme which appeal to the respondent).
5. Has anybody ever convinced/encouraged you to subscribe/renew your member to the NHIS? (Probe for category of persons who influenced respondent [friends, relatives, colleagues at work/school etc.] and the reasons those people gave for asking them to subscribe).

**SECTION 3: Barriers to health insurance subscription**

1. (For subscribers) What is/are the main barriers associated with subscribing to the NHIS?

(For non-subscribers) What are the main factors which serve as barriers to your subscription to the NHIS?

1. How do you consider the quality of drugs you were given the last time you accessed health care from a health facility? (Probe for level of quality ranging from very high quality, high quality, poor quality, very poor quality)
2. For how many minutes/hours did you have to wait in a queue in order to see a doctor the last time you accessed health care from a health facility?
3. How satisfied were you, with the amount of time you stayed in a queue before seeing a doctor the last time you accessed health care? (Probe for the respondent’s level of satisfaction ranging from highly satisfied, satisfied, not satisfied to not at all satisfied).
4. (For subscribers only). How discouraging was attitude of NHIS staff towards you the last time you went to the facility to register/renew you membership?
5. How discouraging was attitude of health care providers towards you the last time you accessed healthcare? (Probe for particular providers whose attitudes discourage respondent and specific attitudes of providers which discourage respondents).

Thank you
